# Supplementary material for: Chewing, dental morphology and wear in tapirs (Tapirus spp.) and a comparison of free-ranging and captive specimens
Source: PLoS One. 2020 Jun 15;15(6):e0234826. doi: 10.1371/journal.pone.0234826 (PMC7295239; doi:10.1371/journal.pone.0234826)
Supplement: Attachment — Submitted filename: Clauss_ReplyLetter_200522.docx [file pone.0234826.s006.docx]

PONE-D-20-05755

Reply to Reviewers

Dear Matthew C. Mihlbachler,

thank you for giving us the opportunity to revise our manuscript and respond to the Reviewers' comments.

Please find attached the revised manuscript with all revised text in grey shading. Our response to specific reviewer comments are given at the bottom of this letter.

We thank you for your time and hope the manuscript now meets the requirements of PLoS One.

Sincerely,

Marcus Clauss

See detailed responses to reviewers down below

Reviewer 1:

Reviewer #1: This is a very interesting and useful piece of basic research with an additional applied aspect that is unlikely to be duplicated any time soon. The work is meticulously executed and documented, the results are clearly presented and the discussion is relevant and lucid. I have only one comment that may, or may not, require minor revision.
Mikael Fortelius

| Reviewer's comment (lines refer to the original submission) | Our response | Changes made in the text (lines refer to the revised version) |
| --- | --- | --- |
| The mesowear method introduced by Fortelius & Solounias was not intended for dental morphologies lacking a distinct buccal wall on the upper teeth. In applying it to tapirs, it might be useful to add a description of how the scoring was done and a discussion of the influence of the bilophodont occlusion on the scores. I suspect that the unusually high scores might reflect some such a circumstance. | Good point, we made this more explicit now than it was in the original manuscript. The description of the score is in detail in the methods. We also added, due to this comment, a sentence in the discussion about the “high” mesowear scores: note that the “high” scroes (when transferred to a numerical score) mean “low occlusal relief”, which is something one would not expect based on a bilophodont occlusion per se. | addition made l. 187: The original mesowear method was explicitly not intended for dental morphologies lacking an ectoloph [2, 33], irrespective of its later applications [reviewed in 34] that also include tapirs [11].  addition made l. 715: Note that one would not expect such a low occlusal relief based on the bilophodont occlusion of tapirs – on the contrary. |

***************************************************************************

Reviewer 2:

Reviewer #2: Overall, I enjoyed reading the manuscript and found it to generally be well done and clearly written. The authors have provided a detailed examination of chewing, tooth anatomy, and tooth wear in tapirs. The findings of the study highlight the orthal movements of the jaws when chewing, which is distinct from other perissodactyls. As a consequence of those movements, the teeth of tapirs have characteristic wear patterns as well. The authors have also compared wear patterns in samples of both wild caught and zoo specimens, revealing generally higher wear in wild populations.
Detailed below I have provided some suggestions for improving the manuscript, along with some points to consider. My main concerns are with the figures, which I feel could be improved in a number of ways that would make the paper easier to read.
Joshua Xavier Samuels

| Reviewer's comment (lines refer to the original submission) | Our response | Changes made in the text (lines refer to the revised version) |
| --- | --- | --- |
| The Supporting Information does provide all of the raw data for the study, but that is not referenced anywhere in the manuscript text. Stating that specimens come from “different European museums” (Page 11, lines 94-95) in the text is not sufficient, particularly when the supporting table just uses museum acronyms. At minimum the authors should list the museums visited (and acronyms) in the Methods, and then provide a reference to the supporting table listing the complete sample details. | very good points | addition made l. 94: The dataset, given *in toto* as S5 Dataset, included 74 individuals  addition made l. 97: dental imprints originating from individual skulls of different European museums, including the Naturhistorisches Museum Basel (NHMB), the National Museums of Scotland Edinburgh (NMS), the Museum für Naturkunde Berlin (ZMB), the Naturmuseum Senckenberg Frankfurt/Main (SMF), the Musée d'Histoire Naturelle Geneva (MHNG), the Zoologisches Museum Hamburg (ZMH), the Natural History Museum London (NHML), the Zoologische Staatssammlung Munich (ZSM), the Staatssammlung für Anthropologie und Paläoanatomie Munich (SAPM), the Musée National d’Histoire Naturelle Paris (MNHN), the Národní Muzeum Prague (NMP), the Naturhistoriska Riksmuseet Stockholm (NRM), the Naturkundemuseum Stuttgart (SMNS), the Naturhistorisches Museum Vienna (NHMW), the Zoologisches Museum Zurich (ZMZ). |
| Page 12, line 117 to Page 13, line 150 and Figure 1 present quantitative measurements taken for this study. While the text descriptions and figure are relatively clear, I think much of this would be well suited to present differently. Presentation of measurements in a table, listing abbreviations and definitions, will make them clear and easy for readers to locate. Figure 1 not only shows measurements themselves, but also a series of relative measures (ratios) derived from them. Restricting the figure to presenting only the actual measurements and showing measurement definitions (both direct and relative measures) in a Table would probably make this all more easily interpretable. | This is a good point. Fig. 1 was modified, a new Table was made, and Table numbering was adjusted. | changes made in Fig. 1, new Table 1 (l. 166) added (not marked) |

| Page 13, lines 154-155 seem to be just randomly placed, rather than flowing directly from the preceding text (“Quantitative measurements”). I would recommend relocating this earlier in the methods, following some of the other basic information regarding the dental formula of tapirs (Page 12, line 114). | makes sense! | change made l. 124: The eruption sequence of permanent teeth in tapirs was assumed as M1 – (P1-P3) – (P4, M2) – M3 [following 31]. |
| --- | --- | --- |
| The Methods on Page 13, line 158 to Page 15, line 191, Results Page 23, lines 420-421, and Table 2 describe derivation of macrowear and mesowear scores. Here, the authors have developed a scoring system for wear stages of individual teeth. A master’s thesis by Gibson (2011) looked at the population structure of a large sample of fossil tapirs, and developed a method for roughly aging tapirs into 7 categories based on tooth eruption and wear stages. Comparison of those classes to the macrowear based age classes developed here would be interesting. Gibson, M.L., 2011. Population Structure Based on Age-Class Distribution of Tapirus polkensis from the Gray Fossil Site Tennessee. MS Thesis. East Tennessee State University.  Page 34, lines 647-654 discuss determination of tapir age by wear, Gibson’s (2011) study of tooth eruption and wear mentioned above would be directly relevant to this section. | This is a good point – we had not been aware of this thesis. We had cited the major source the thesis derived its system from (Maffei) already, and had already discussed how the one age information we had did not match that of Maffei; the thesis was now included in that discussion. This also led us to a similar system, published in 2009, which we also cite now.  We mention in the methods now that a difference between the previous systems and ours is – somehow – that we go beyond calling wear “slight” or “heavy” but actually describe the occlusal surface of the individual stages with criteria that are, at least a bit, less subjective. | addition made l. 173: Macrowear scoring systems, based on verbal descriptions (from no wear to slight/little wear, moderate wear, to heavy wear) for tapirs have developed before [32-34]; we aimed at providing anatomy-based descriptions for these stages.  additions made l. 676: Maffei [32] and Gibson [33] developed keys to determine tapirs ages by tooth wear of the right lower mandibula based on hunted free-ranging individuals. As we mostly had no information about age from our specimens, we cannot compare our material to his. The only skull with identified age was labelled as 10 years old and had non-occluding third molars, which is contradictory to the age keys of Maffei [32] and Gibson [33] (wear on M3 present at 8 years). We hope that the more detailed anatomical description of macrowear stages provided in Table 3, and the possibility of quantifying wear using objective measurements (RLD, RLL, RLW), will facilitate more detailed future studies on relationships of tapir tooth wear and age. |
| The results are dominated by quite a few Figures, which are each described as presenting correlations between variables studied. Rather than a large number of bivariate plots in the text, I wonder if this content could be presented in a more straightforward fashion using tables, and then potentially providing plots in supporting material. For example, I think Figures 13-15, and 17 could all be presented as tables | This is something we had debated/discussed quite a bit before the original submission. The choice of PLoS One as the primary journal for submission was largely dominated by our impression that in this journal, the number of figures would not be considered prohibitive. We played different versions – tables, supplementary files with graphs – and we decided on the figures in the end. We would really like to keep this. In particular, some of us really felt that shape and impressions of the scatter plots are more instructive than tables. We would be grateful if this was permitted. | we would like not to change this |
| Figures 4, 6-10, 12-14, etc. would be more readable with some enlargement of fonts. | Point taken, ALL figures re-done. Also, axis scaling was made consistent across graphs, so that size differences between the species are implicitly shown now. | All figures re-done as suggested (not marked). |

| Also, in Figure 4 there are two distinct gradients used to indicate tooth position in A and B. I find the direct transition of tones in Fig. 4B to be more easily interpreted, and suggest changing A to be a similar sort of transition in grayscale tones. | Point taken, figures re-done. | Figure re-done as suggested (not marked). |
| --- | --- | --- |
| Figure 5 would be more appropriately included earlier in the Methods (lines 109-112 would work), and can be referred to in the Results as well. | Point taken, figure re-arranged and all figure numbering revised. | Figure placement changed as suggested (not marked). |
| Figures 6 through 10 include a line, but that is not defined anywhere. I assume it is just showing equal proportions, but it should be clear that this is not some sort of regression line or something else. | Point taken, explanation added to the corresponding legends. | addition made in all corresponding figure legends: The line denotes y=x. |
| Page 28, line 508 to page 29, line 510 discusses the lophodont pattern and inferred orthal movement of the tapirs. Many mammals with some form of lophodont occlusal morphology (including loxodont, ptychodont, and selenolophodont teeth) have primary orientation of those folds/ridges perpendicular to the primary direction of jaw movement (Ungar 2010). Proboscideans (loxodont), rodents (many lophodont or ptychodont lineages), and some marsupials (like kangaroos and wombats) show mediolaterally oriented lophs on their teeth and a primary orthal (propalinal) movement of the jaws when chewing. Mentioning those sorts of common patterns helps support the interpretations here. Ungar, P.S., 2010. Mammal teeth: origin, evolution, and diversity. JHU Press. | ok, we added some of this, also stressing the difference: there is no propalinal movement in tapirs in our view. | addition made l. 524: Transferring from the interplay of dental anatomy and chewing direction from other species such as elephants, rodents or macropods [4], the mediolateral orientation of the lophs of tapir cheek teeth would lead to the expectation of propalinal rather than lateral chewing movements.  addition made l. 529: A propalinal movement was not observed.  addition made l. 549: Our second line of circumstantial evidence against distinct lateral or propalinal chewing movements comes from the – admittedly subjective – manipulation of a tapir skull  addition made l. 556: Manipulation of the skull imitating propalinal chewing was not possible. |
| Page 29, line 531 to page 30, 537 discusses the size of the temporalis muscle and sagittal crest. Outside of ungulates, there are many other herbivorous mammals with large and prominent temporalis and sagittal crest, including many rodents like beavers and chisel tooth digging burrowers (Samuels 2009) and primates like Gorillas. | good point, addition made | addition made l. 562: A sagittal crest is prominent in rodents that require a strong incisor bites, such as in chisel-tooth digging burrowers or species that are particularly lignivorous such as beavers [46]. |
| Figure 23 caption, the word comparison is redundant at the start of the sentence. | indeed, thank you | deletion made (not marked) |

| Page 35, line 678 to Page 37, line 724 discuss the mesowear of tapirs, and the fact Tapirus bairdii has a greater mesowear than is typical of other brachydont species. Rather than the methodology being compromised, as mentioned in lines 694-695, it is important to remember mesowear reflects attrition of the tooth over a substantial portion of the animal’s life (Fortelius and Solounias 2000, Davis and Pineda-Munoz 2016), and thus could potentially be a consequence of multiple factors. What the authors mention in lines 721 to 724 is what I suspect is the most important part of this and should be expanded upon. While grazing or feeding on grit-covered plants in relatively arid habitats may be the typical way teeth wear in herbivorous mammals, I suspect consumption of fruits and nuts with hard seeds or pits could also potentially lead to higher attrition of the teeth in wild tapir populations. Though such foods might not be the bulk of the diet in a population, they could still have a substantial impact on tooth wear. Additionally, the actual ages of individuals in these relatively small samples is unknown. If the T. bairdii sample had individuals of more advanced age than the other two tapir species (or other previously published brachydont browsers), they would be expected to have relatively more advanced wear. | We mention this as one alternative view now, although some of us (esp. MC) believe that the concept of “hard items” leading to wear in the described manner is a classic misleading rhetoric without substance. Also, the wording “hard seeds could lead to attrition” is a contradiction in itself where “attrition” is defined as tooth-to-tooth contact – why should seeds increase that? Would one not expect less force during chewing when hitting on hard objects (imagine eating an olive or a cherry – does that induce more forceful chewing, or more careful chewing?).  Note that age was indirectly excluded by ‘counting’ mesowear only for specimens within macrowear stages 1-3. | addition made l. 739: Whether the higher mesowear scores in *T. terrestris* and *T. bairdii* can be linked to the ingestion of fruits with hard seeds appears dubious to us, as we are not aware of reports of seed-cracking chewing behaviour in tapirs, and because the senior author observed intact large seeds in the stomach content and faeces of free-ranging *T. terrestris* investigated for a digestion study [52]. However, we note that the effect of large seeds on mesowear has not been investigated experimentally so far. |
| --- | --- | --- |

**************************************************************************
